# Post–endoscopic retrograde cholangiopancreatography pancreatitis prophylaxis: a stratified assessment for evaluation of risk approach

**DOI:** 10.1016/j.igie.2025.10.009

**Published:** 2025-10-17

**Authors:** David S. Braun, Prashant S. Kedia, Mingyang Cui, Paul R. Tarnasky

**Affiliations:** 1 Department of Gastroenterology, Methodist Dallas Medical Center, Dallas, Texas, USA; 2 Digestive Disease Institute, Methodist Dallas Medical Center, Dallas, Texas, USA; 3 Clinical Research Institute, Methodist Health System, Dallas, Texas, USA

## Abstract

**Background and Aims:**

The risk of post–endoscopic retrograde cholangiopancreatography (ERCP) pancreatitis (PEP) is influenced by patient and procedure-related factors. Strategies to decrease PEP are of great importance. This study aimed to determine if PEP prophylaxis (PEPX) could be avoided in patients in whom high-risk procedure-related factors are avoided as guided by a Stratified Assessment for Evaluation of Risk (SAFER) approach. SAFER is a dynamic process whereby procedure-related factors direct PEPX strategies.

**Methods:**

A prospective database of consecutive ERCPs was analyzed. Data regarding demographics, procedure indications, previous ERCP attempts, cannulation techniques, PEPX received, and adverse events were collected. Each ERCP was classified for PEP risk pre- and post-ERCP based on patient factors, procedure indications, findings, and techniques. PEP incidence was assessed with or without PEPX after stratifying risk using a SAFER approach.

**Results:**

From a cohort of 3006 procedures, PEP incidence without any PEPX in all patients with low post-ERCP risk (n = 2096) was 0.2%. Independent of PEPX, there was no significant difference in the incidence of PEP between procedures with both low pre- and post-ERCP risk and those with high pre-ERCP risk but low post-ERCP risk.

**Conclusions:**

To influence PEPX decisions, procedure-related factors may be more important than patient-related PEP risk factors. A SAFER approach suggests that low-risk post-ERCP patients do not need PEPX, although further prospective studies are needed.

## Introduction

The most common and feared adverse event of endoscopic retrograde cholangiopancreatography (ERCP) is post-ERCP pancreatitis (PEP). The incidence of PEP is influenced by operator-, patient-, and procedure-related risk factors, and risk escalates when multiple factors are present.^1^ Overall, the average PEP incidence ranges from 3% to 10%.^2^, ^3^, ^4^ In prospective randomized controlled trials (RCTs) evaluating PEP prevention methods, the PEP rate in placebo groups was 10% to 13%.^5^, ^6^, ^7^ Despite decades of research and proven methods for PEP prophylaxis (PEPX), the incidence of PEP is increasing.^3^ A national inpatient cohort study reported an increase in PEP risk from 12% in 2007 to 16% in 2016.^8^ Another study reported an overall PEP rate of 4.5% for inpatient ERCP procedures, but it increased by 15% from 2011 to 2017.^9^

Since the landmark RCT by Elmunzer et al,^10^ rectal nonsteroidal anti-inflammatory drugs (RNSAIDs) have been widely recommended for PEPX for all patients.^11^^,^^12^ A proven strategy to reduce PEP in high-risk patients is ensuring pancreatic duct (PD) drainage with prophylactic pancreatic stenting (PPS).^13^ The increased use of RNSAIDs has correlated with decreased PPS utilization.^13^^,^^14^ Both PPS and RNSAIDs are reported to be cost-effective for reducing PEP.^15^ However, a controversy remains as to the preferred PEPX method, and full adoption of PEPX practices is lacking.^16^

We tested the concept that PEP risk assessment is dynamic, specifically, that a patient's PEP risk may change from low to high risk (or vice versa) after cannulation and subsequent techniques. Our hypothesis was 2-fold. First, low-risk patients do not need PEPX unless their risk level increases to high risk by procedural factors (eg, difficult cannulation). Second, high-risk pre-ERCP patients do not need PEPX if procedural factors that increase risk (eg, pancreatography) are avoided. We assessed the potential of a Stratified Assessment for Evaluation of Risk (SAFER) approach to guide decisions regarding PEPX.

## Methods

### Study design and setting

A prospective database (January 2012–December 2023) was maintained of consecutive ERCPs performed over 12 years by 1 endoscopist (P.R.T.) at Methodist Dallas Medical Center (Dallas, Tex, USA), a tertiary referral center with an annual ERCP volume >500 ERCPs. Data were analyzed to assess PEP incidence according to risk factors. The study was approved by the Methodist Health System Institutional Review Board (3046.GID.2024.D).

### Participants and variables

The data from consecutive patients who underwent ERCP were reviewed. Reasons for excluded procedures (eg, cannulation not attempted, aberrant anatomy) are detailed in Figure 1. Baseline data collected included demographics, procedure indications, previous ERCP attempt, pertinent surgical history, and PEPX. Prior ERCP attempts were categorized by location as outside institution, other (home institution by a different provider), or here (home institution by the primary provider) and by cannulation outcome (successful or failed). Patients with prior ERCP may or may not have undergone previous endotherapy.

**Figure 1:**
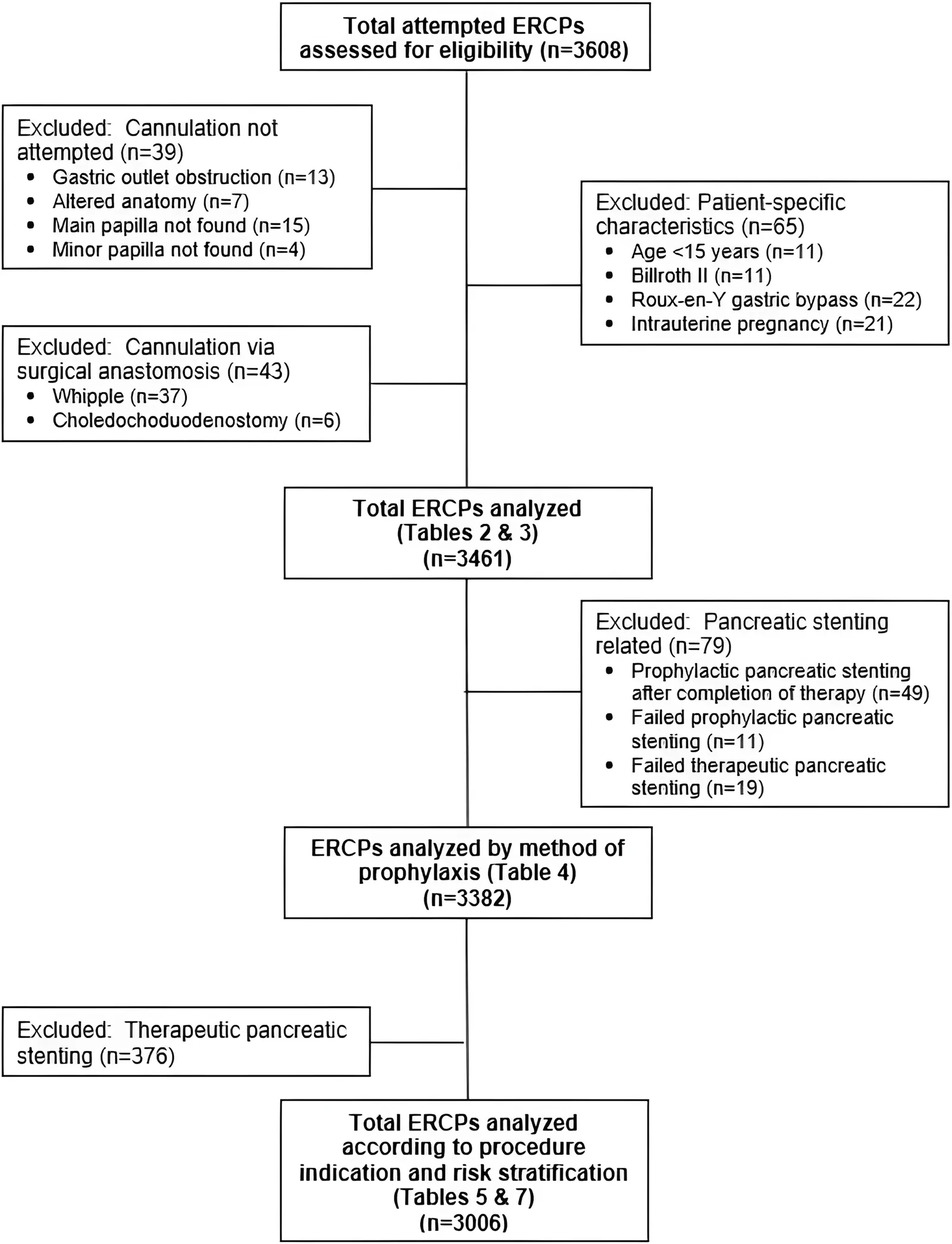
Flow diagram of the study patient cohort. *ERCP*, Endoscopic retrograde cholangiopancreatography.

Patients were considered to have a native main papilla if there was no prior biliary and/or pancreatic sphincterotomy, no current indwelling stent, or no prior accessory papilla therapy and the intention was for therapy thereof. Patients with prior failed cannulation were considered to have a native papilla. However, those with a prior PD stent in place (even if the prior biliary cannulation attempts failed) and/or a prior failed precut sphincterotomy were not considered to have a native papilla.

ERCP indications were categorized as biliary, pancreatic, or both. Examples for both indications included ampullary neoplasia, idiopathic pancreatitis with increased serum liver tests, and selected patients with postcholecystectomy abdominal pain related to suspected sphincter of Oddi dysfunction (SOD). Procedure-related data collected included method of sedation, endotherapy performed, duct of Santorini patency (as observed on fluoroscopy if/when pancreatography was performed), adverse events, and disposition (overnight observation, inpatient, or discharge). Whether pancreatography was attempted (successful or failed), and whether the PD was cannulated with a guidewire (successful or failed) or not attempted (none or inadvertent) was also recorded.

Patients admitted overnight and inpatients were observed for potential adverse events. Discharged patients had follow-up visits with the endoscopist and/or were contacted by nursing staff within 1 week after ERCP to assess for potential adverse events. All adverse events, including PEP, were diagnosed and graded according to well-established standard definitions.^17^

### Procedures

Technical strategies included operator-controlled guidewire cannulation, avoidance of pancreatography for biliary indications only, and precut sphincterotomy for difficult cannulation (eg, after ≥3 inadvertent PD guidewire cannulations). Pancreatography was limited to pancreatic indications or to confirm proper PD guidewire position; contrast injections were controlled by the operator. Sphincterotomies were performed using monopolar endocut-forced coagulation. A sphincterotome (Boston Scientific, Marlborough, Mass, USA) with 20-mm cutting wire was used for over-the-wire sphincterotomies. A needle-knife papillotome was used for precut sphincterotomies.

Trainees were involved to some extent in nearly all procedures. Guidewires were controlled by the attending endoscopist in all cases involving cannulation of a native papilla. Trainees did not participate directly with high-risk procedures (eg, patients with pancreas divisum or suspected SOD).

For PPS, 4F × 9-cm, single-pigtail pancreatic stents without a proximal flap were the preferred stents, for example, when a precut sphincterotomy was performed after inadvertent PD guidewire access. Short (≤3-cm) 5F stents were used less often, for example, when there was an ansa loop of the PD. RNSAIDs were administered during or after ERCP in selected patients at high risk for PEP, for example, inadvertent pancreatography without PD access, failed PPS, or if mandated by a research protocol. Therapeutic PD stents (TxPDSs) were placed if required for therapy of relapsing chronic pancreatitis (eg, strictures). Some procedures with pancreatic stenting were excluded from PEP risk analyses (Fig. 1).

### Risk assessment

Pre-ERCP (patient-related) and post-ERCP (procedure-related) PEP risk for each ERCP was classified according to indications, findings, and procedural techniques (Table 1). Some patients who would normally be classified as high pre-ERCP PEP risk were considered low risk in some circumstances. All patients with a prior sphincterotomy and biliary indication only were considered as low pre-ERCP risk. For example, patients with a history of PEP were deemed low risk if they had a prior biliary sphincterotomy and had only a biliary indication for ERCP. Also, patients with pancreas divisum were considered low risk if undergoing ERCP for only biliary indications (eg, ampullectomy). Some patients were considered high risk at their first ERCP but low risk on subsequent ERCPs (eg, those with a history of acute pancreatitis with distal PD transection found on prior ERCP). Also, patients with a pre-ERCP high-risk indication were converted to low-risk post-ERCP if pancreatography was avoided.

**Table 1:** Classification of PEP risk

PEP risk	Before ERCP	During and after ERCP
Low	Pancreatic cancer	Avoided pancreatography
	Chronic calcific pancreatitis	Patent duct of Santorini
	Known disconnected pancreatic duct syndrome	Disconnected pancreatic duct syndrome
	Biliary indication only	Ventral pancreas and only biliary intervention∗
	Prior sphincterotomy	
	Indwelling stent	
High	History acute pancreatitis	Difficult cannulation†
	History of PEP	Inadvertent pancreatography
	Known common channel observed on prior imaging (eg, MRCP, intraoperative cholangiography)	Ampullectomy
	Primary sclerosing cholangitis with native papilla	Precut sphincterotomy
	Suspected sphincter of Oddi dysfunction	Accessory (minor) sphincterotomy
		Pancreatoscopy
		Pancreatic brush cytology
		Fully covered metal biliary stent without sphincterotomy and normal pancreas

Post-ERCP pancreatitis is defined as new clinical pancreatitis more than 24 hours after procedures and requiring admission or prolongation of admission.^17^

*ERCP*, Endoscopic retrograde cholangiopancreatography; *MRCP*, magnetic resonance cholangiopancreatography; *PEP*, post–endoscopic retrograde cholangiopancreatography pancreatitis.

∗ Cannulation of the main papilla, which may include injection of the ventral duct but does not access the main pancreatic duct, for example, pancreas divisum.

† Repeated inadvertent pancreatic duct cannulations with catheter and/or guidewire, double-wire cannulation technique.

An otherwise low-risk patient (eg, bile duct stone) would be converted to high risk if there was inadvertent pancreatography (eg, common channel filling). Also, a low-risk patient with chronic calcific pancreatitis would be converted to high risk if pancreatoscopy was performed. Patients with low-risk indications were deemed high-risk post-ERCP in a setting of inadvertent pancreatography and/or a difficult cannulation requiring precut. If any of the post-ERCP high-risk criteria were met (Table 1), they were considered high postcannulation risk for PEP.

### Statistical methods

STATA, version 16.1 (StataCorp LLC, College Station, Tex, USA), was used for statistical analyses. Quantitative data were presented as median with interquartile range or mean with standard deviation, whereas categorical variables were presented as frequency with percentage. Fisher exact and Wilcoxon rank-sum (Mann-Whitney U) tests were used as indicated. Logistic regressions were used for multivariate analyses. *P* values <.05 were considered statistically significant.

## Results

### Cohort demographics

A total of 3608 ERCP procedures were attempted during the 12-year study. After exclusion of 147 ERCPs, 3461 ERCP procedures (57.2% female; mean [standard deviation] age: 57.6 [17.6] years) were included in the analyses (Fig. 1).

Procedures on patients with prior pertinent surgeries included cholecystectomy (n = 1860) and orthotopic liver transplant (n = 244). Procedures were mostly for biliary indications (n = 2269, 66.0%). Some procedures had primary pancreatic indications (n = 871, 25.2%); the remainder had both biliary and pancreatic indications (n = 321, 9.3%) (Table 2). The most common primary ERCP indications included repeat endotherapy (n = 1247), abnormal liver function test results and/or jaundice (n = 877), therapy for recurrent and/or adverse events of acute pancreatitis (n= 311), unexplained postcholecystectomy pain (n = 245), unexplained acute pancreatitis (n = 216), and abnormal intraoperative cholangiogram (n = 189). A total of 134 procedures were performed with the goal of accessory papilla therapy.

**Table 2:** Indications, patient status, and disposition of study cohort (n = 3461)

Indications for ERCP	Patient status at time of ERCP		Disposition after ERCP		
	Outpatient	Inpatient∗	Discharge	Admit for observation	Inpatient
Biliary	1261	1008	1233	30	1006
Pancreatic	751	120	686	61	124
Both	266	55	232	31	58
Totals	2278	1183	2151	122	1188

*ERCP*, Endoscopic retrograde cholangiopancreatography.

∗ Includes 50 patients transferred from another institution for ERCP and then transferred back to that institution following the procedure.

### Adverse events

The overall adverse event rate was 2.7% (95 of 3461) (Table 3). Most (n = 61, 64%) occurred in patients who were discharged after ERCP. The remainder of overall adverse events occurred in patients admitted for observation (n = 19) or in patients already admitted (n = 15). A total of 131 procedures (3.8%) were performed in patients with a history of prior PEP. The prior PEP was most commonly (74.0%) after an outside ERCP (n = 79) or an outside failed ERCP (n = 18). The incidence of PEP was 1.5% in our cohort (n = 51); overall 1% had mild PEP (n = 34) and 0.5% had moderately severe PEP (n = 17). No patients experienced severe PEP. In the overall cohort (n = 3461), mean age was significantly lower for those that developed PEP (48.5 [15.7] years) versus those that did not (57.9 [17.5] years; *P* < .001). With age as a potential confounder, there was no significant difference in PEP incidence between male (1%) and female (1.8%). With age and gender as potential confounders, PEP incidence was significantly higher after procedures with pancreatic (3.3%) or both biliary and pancreatic (3.9%) versus intended biliary cannulation only (0.5%; *P* < .001; adjusted odds ratio [aOR], 7.37; 95% confidence interval [CI], 3.70-14.69). With age, gender, and intended cannulation as potential confounders, PEP was significantly more common with a native papilla (2.1%) versus those without (1.0%; *P* = .018; aOR, 2.03; 95% CI, 1.13-3.63).

**Table 3:** Overall adverse events of study cohort (n = 3461)

Previous ERCP	Any previous PEP		Native papilla		Adverse events according to severity
	Yes	No	Yes	No	
No, n = 1264	NA	NA	1264	NA	Mild PEP: 19; 6 of 19 delayed attack Moderate PEP: 7; 2 of 7 delayed attack Pain: 5 mild Bleed: 4 moderate, 5 severe Cholangitis: 1 mild Respiratory failure: 1 Mortality: 3∗
Home institution, n = 1318	61	1257	6	1312	Mild PEP: 5 Moderate PEP: 6; 2 of 6 delayed attack Bleed: 2 mild; 1 of 2 delayed Cholangitis: 5 mild; 1 of 5 delayed Hemobilia: 1 mild, 1 moderate Pain: 5 mild Perforation: 1 severe Duct perforation with delayed stent migration: 1 severe
Failed at home institution, n = 16	3	13	3	13	0
Home institution by another provider, n = 101	1	100	3	98	0
Failed at home institution by another provider, n = 11	0	11	4	7	0
Outside institution, n = 542	53	489	35	507	Mild PEP: 8; 1 of 8 delayed Moderate PEP: 1 delayed Pain: 3 mild Bleed: 1 moderate delayed Cholangitis: 1 mild Seizure: 1 mild Perforation: 1 moderate
Failed at outside institution, n = 209	13	196	173	36	Mild PEP: 2; 1 of 2 delayed Moderate PEP: 3 Bleed: 1 mild delayed Perforation: 1 severe delayed

All post-ERCP adverse events defined by standard definitions.^17^

Delayed is defined as onset more than 24 hours after ERCP.

*ERCP*, Endoscopic retrograde cholangiopancreatography; *PEP*, post–endoscopic retrograde cholangiopancreatography pancreatitis; *NA*, not applicable.

∗ Three patients died 24 to 72 hours after ERCP; all had malignant biliary obstruction.

### Methods of prophylaxis

Pancreatic stenting for PEPX included both small-caliber stents without proximal flaps, designed for self-migration (ie, PPS), and TxPDSs (n = 376) for patients with a PD pathology (eg, stones, strictures, and/or leaks). Among the 11 procedures with failed PPS (7 with pancreas divisum, 3 with ampullary neoplasia, and 1 with SOD), mild PEP occurred in 3 cases. Of those, early-study patients (up to 2016) were not administered RNSAIDs; 1 of 4 had mild PEP. From 2017 on, 1 refused participation in an RNSAID trial, but the remaining 6 were all treated with RSNAIDs for PEPX; 2 of 7 had mild PEP. In the 19 procedures with failed TxPDSs, mild PEP was noted in 1 case; an endoscopic ultrasonography–guided cyst drainage was performed during the same session. Another 49 procedures involved PPS to ensure drainage after therapy for chronic pancreatitis; none were complicated by PEP.

There was no significant difference in the incidence of PEP after procedures with only RNSAIDs (1.0%), only PPS (2.4%), or only TxPDSs (1.9%) (Table 4). Patients who received both RNSAIDs and PPS were significantly more likely to develop PEP than those patients who received PPS alone (11.9% vs 2.4%; aOR, 5.19; 95% CI, 2.44-11.04; *P* = .007). Procedures without PEPX were associated with significantly less PEP than those with PPS alone (0.4% vs 2.4%; aOR, 3.89; 95% CI, 1.45-10.40; *P* < .001) and those with both PPS and RNSAIDs (0.4% vs 11.9%; aOR, 20.15; 95% CI, 7.34-55.35; *P* < .001).

**Table 4:** PEP severity according to method of prophylaxis (n = 3382)

PEP severity	Method of prophylaxis, n (%)					
	None (n = 2150)∗	PPS (n = 630)	TxPDSs (n = 365)	RNSAIDs alone (n = 100)	PPS + RNSAIDs (n = 126)	TxPDSs + RNSAIDs (n = 11)
Overall (n = 47)	8 (0.4)†^,^‡	15 (2.4)	7 (1.9)	1 (1.0)	15 (11.9)	1 (9.1)
Mild (n = 30)	4 (0.2)	10 (1.6)	6 (1.6)	1 (1.0)	8 (6.3)	1 (9.1)
Moderate (n = 17)	4 (0.2)	5 (0.8)	1 (0.3)	0 (0)	7 (5.6)	0 (0)

*ERCP*, Endoscopic retrograde cholangiopancreatography; *PEP*, post–endoscopic retrograde cholangiopancreatography pancreatitis; *PPS*, prophylactic pancreatic stenting; *RNSAID*, rectal nonsteroidal anti-inflammatory drug; *TxPDS*, therapeutic pancreatic duct stent.

∗ Includes 4 procedures with intended PPS but stent migrated out during procedure; none of them were complicated by PEP.

† Procedures with no PEP prophylaxis were associated with significantly less PEP compared to both PPS alone and PPS + RNSAIDs (*P* < .001, adjusted), considered age, sex, native papilla, and intended biliary/pancreatic/both as potential confounding variables.

‡ Procedures with no PEP prophylaxis were associated with significantly less PEP compared to TxPDSs + RNSAIDs (*P* = .016, adjusted), considered age, sex, native papilla, and intended biliary/pancreatic/both as potential confounding variables.

The incidence of PEP and the methods of PEPX for the final study cohort (n = 3006) were stratified by procedure indication and papilla status (Table 5). PEP incidence was significantly lower in patients undergoing ERCP for biliary-only indications (0.5%) than in those with pancreatic-only (4.1%) or both biliary and pancreatic indications (3.2%; *P* < .001). Patients with a native papilla had a significantly higher incidence of PEP (2.0%) than those with prior papilla therapy (0.7%; *P* = .016; aOR, 2.4; 95% CI, 1.18-4.87). With age, gender, PEPX, papilla status (eg, native), and intended cannulation as potential confounders, PEP was significantly less common after procedures with no pancreatography (0.3%) versus those with a nonpatent minor papilla (6.1%) or unsure, that is, when patency of the minor papilla could not be confirmed on fluoroscopy (2.0%; *P* ≤ .001; aOR, 21.09; 95% CI, 5.96-74.62).

**Table 5:** PEP incidence and prophylaxis based on patient and papilla-related factors (n = 3006)

Patient- or procedure-related factors	Total PEP, n/n (%)	PEP according to prophylaxis, n/n (%)			
		None	PPS	RNSAIDs	PPS + RNSAIDs
Indications					
Biliary (n = 2265)	11/2265 (0.5) 8 mild 3 moderate	7/1975 (0.4)	1/178 (0.6)	1/84 (1.2)	2/28 (7.1)
Pancreatic (n = 459)	19/459 (4.1) 8 mild 11 moderate	1/80 (1.3)	7/299 (2.3)	0/12 No PEP	11/68 (16.2)
Both (n = 282)	9/282 (3.2) 7 mild 2 moderate	0/95 No PEP	7/153 (4.6)	0/4 No PEP	2/30 (6.7)
Total PEP for all indications	39/3006 (1.3)	8/2150 (0.4)	15/630 (2.4)	1/100 (1)	15/126 (11.9)
Mild severity	23/3006 (0.8)	4/2150 (1.9)	10/630 (1.6)	1/100 (1)	8/126 (6.3)
Moderate severity	16/3006 (0.5)	4/2150 (1.9)	5/630 (0.8)	None	7/126 (5.6)
Intended papilla status					
Native (n = 1366) Includes 74 intended accessory papilla therapy	27/1366 (2.0) 18 mild 9 moderate	5/855 (0.6)	12/350 (3.4)	1/73 (1.4)	9/88 (10.2)
Prior therapy (n = 1640) Includes 60 intended accessory papilla therapy	12/1640 (0.7) 5 mild 7 moderate	3/1295 (0.2)	3/280 (1.1)	0/27 No PEP	6/38 (15.8)
Minor papilla patency: excluding pancreas divisum or accessory papilla therapy (n = 141)					
No pancreatography, NA (n = 2000)	5/2000 (0.3) 4 mild 1 moderate	5/1933 (0.3)	None	0/67 No PEP	None
Patent (n = 46)	None	0/11 No PEP	0/25 No PEP	0/3 No PEP	0/7 No PEP
Not patent (n = 313)	19/313 (6.1) 11 mild 8 moderate	2/43 (4.7)	8/214 (3.7)	None	9/56 (16.1)
Unsure (n = 506)	10/506 (2.0) 5 mild 5 moderate	1/141 (0.7)	5/300 (1.7)	1/29 (3.4)	3/36 (8.3)

*CI,* Confidence interval; *ERCP*, endoscopic retrograde cholangiopancreatography; *NA*, not applicable; *OR*, odds ratio; *PEP*, post–endoscopic retrograde cholangiopancreatography pancreatitis; *PPS*, prophylactic pancreatic stenting; *RNSAID*, rectal nonsteroidal anti-inflammatory drug.

Comparing procedure indications, PEP rate was significantly higher in patients with pancreatic than in patients with biliary (*P* < .001; adjusted OR, 8.67; 95% CI, 3.60-20.93). PEP rate was significantly higher in patients with both pancreatic and biliary than in patients with biliary (*P* < .001; adjusted OR, 6.95; 95% CI, 2.62-18.46). Age, sex, and native papilla were considered as potential confounders.

Comparing procedures with native papilla versus prior therapy, PEP rate was significantly higher in patients with native papilla than in patients without native papilla (*P* = .016; adjusted OR, 2.4; 95% CI, 1.18-4.87). Age, sex, and biliary/pancreatic/both were considered as potential confounders.

For PEP according to prophylaxis, PEP rate was significantly higher in PPS than in none (*P* = .007; adjusted OR, 3.89; 95% CI, 1.45-10.40). PEP rate was significantly higher in RNSAIDs + PPS than in none (*P* < .001; adjusted OR, 20.15; 95% CI, 7.34-55.35). PEP rate was significantly higher in RNSAIDs + PPS than in PPS (*P* < .001; adjusted OR, 5.19; 95% CI, 2.44-11.04). PEP rate was significantly higher in RNSAIDs + PPS than in RNSAIDs (*P* = .023; adjusted OR, 11.17; 95% CI, 1.39-89.92). Age, sex, native papilla, and biliary/pancreatic/both were considered as potential confounders.

Regarding minor papilla patency, PEP rate was significantly higher in patients with not patent than in NA (*P* < .001; adjusted OR, 21.09; 95% CI, 5.96-74.62). PEP rate was significantly higher in unsure than in NA (*P* = .001; adjusted OR, 7.77; 95% CI, 2.23-27.09). PEP rate was significantly higher in not patent than in unsure (*P* = .016; adjusted OR, 2.72; 95% CI, 1.20-6.12). Age, sex, native papilla and biliary/pancreatic/both were considered as potential confounders.

Among procedures with only biliary indications (Table 6), the PEP rate was significantly higher (3%) in patients with inadvertent pancreatography (includes those with inadvertent PD guidewire) than in patients without inadvertent PD manipulation (0.2%; *P* < .001; aOR, 22.69; 95% CI, 4.82-106.84). However, there was no significant difference in PEP incidence between procedures without inadvertent PD manipulation and those with inadvertent PD guidewire cannulation only. In addition, there was no significant difference in PEP incidence between procedures without inadvertent PD manipulation and those with precut sphincterotomy.

**Table 6:** PEP incidence and prophylaxis based on procedure-related factors for procedures with only biliary indications (n = 2265)

Inadvertent main pancreatic duct manipulation	Total PEP, n (%)	PEP prophylaxis, n (%)			
		None	PPS	RNSAIDs	Both
None (n = 1903) Includes ventral duct with pancreas divisum (n = 11)	4 (0.2) 4 mild	1843 (96.8) 5 (0.3)	0	61 (3.2) 0	0
Pancreatic guidewire only (n = 50)	0	47 (94) 0	3 (6) 0	0	0
Pancreatography (n = 100) Includes 18 with common channel filling of pancreatic duct	4 (4) 2 mild 2 moderate	70 (70.0) 2 (2.9)	5 (5.0) 0	20 (20.0) 1 (5)	5 (5.0) 1 (20)
Pancreatic guidewire + pancreatography (n = 34)	0	4 (11.8) 0	24 (70.6) 0	2 (5.9) 0	4 (11.8) 0
Precut sphincterotomy (n = 177) Includes 11 with failed biliary access after precut	2 (1.1) 2 mild	11 (6.2) 0	146 (82.5) 1 (0.7)	1 (0.5) 0	19 (10.7) 1 (5.3)

PEP rate was significantly higher in patients with inadvertent pancreatography (includes those with inadvertent pancreatic duct guidewire) than in patients without inadvertent pancreatic duct manipulation (*P* < .001; adjusted OR, 22.69; 95% CI, 4.82-106.84). Age, sex, and native papilla were considered as potential confounders.

*CI,* Confidence interval; *ERCP*, endoscopic retrograde cholangiopancreatography; *OR*, odds ratio; *PEP*, post–endoscopic retrograde cholangiopancreatography; *PPS*, prophylactic pancreatic stenting; *RNSAID*, rectal nonsteroidal anti-inflammatory drug.

One procedure involved inadvertent cannulation of pancreatic duct with manometry catheter only without pancreatography and developed moderate PEP.

### Stratified post-ERCP risk analysis

Of 3006 procedures, 1853 (61.6%) had low pre- and post-ERCP PEP risk (Table 7). For all procedures classified as low post-ERCP risk (n = 2173), the overall incidence of PEP was 0.2%. A total of 833 procedures (27.7%) were categorized as high post-ERCP risk. The incidence of PEP in the low-low, high-low, low-high, and high-high PEP risk groups was 0.2% (n = 3), 0.6% (n = 2), 1.3% (n = 3), and 5.1% (n = 31), respectively. By multivariate analyses (using age, sex, native papilla, and indications as potential confounding variables), the high-high PEP risk group was associated with significantly higher PEP compared with all other risk groups.

**Table 7:** Incidence of PEP according to the stratified risk assessment and prophylaxis methods (n = 3006)

PEPX	Pre- and postcannulation PEP risk, n/n (%)			
	Low-low (n = 1853)	High-low (n = 320)	Low-high (n = 224)	High-high (n = 609)
None (n = 2150)	3/1849 (0.2) 3 mild	2/247 (0.8) 2 moderate∗	2/40 (5.0) 2 moderate	1/14 (7.1) 1 mild
Any PEPX (n = 856)	0/4	0/73	1/184 (0.5) 1 mild	30/595 (5.0) 18 mild 12 moderate
RNSAIDs (n = 100)	0/1	0/69	1/20 (5.0) 1 mild	0/10
PPS (n = 630)	0/3	0/4	0/151	15/472 (3.2)† 10 mild 5 moderate
RNSAIDs + PPS (n = 126)	None	None	0/13	15/113 (13.3) 8 mild 7 moderate

*CI,* Confidence interval; *ERCP*, endoscopic retrograde cholangiopancreatography; *OR*, odds ratio; *PEP*, post–endoscopic retrograde cholangiopancreatography; *PEPX*, post-ERCP pancreatitis prophylaxis; *PPS*, prophylactic pancreatic duct stenting; *RNSAIDs*, rectal nonsteroidal anti-inflammatory drugs.

∗ 1 associated with inadvertent pancreatic duct cannulation with manometry catheter, 1 had endoscopic ultrasonography with fine-needle aspiration at same setting.

† Significantly lower PEP compared to RNSAIDs + PPS (*P* < .001; adjusted OR, 4.08; 95% CI, 1.89-8.82), adding age, sex, native papilla, and biliary/pancreatic/both as confounding variables.

No significant difference in PEP incidence was observed between the low-low and high-low PEP groups who did not receive PEPX (0.2% vs 0.8%; *P* = .374). Also, among patients with both low pre- and post-ERCP risk, there was no significant difference in PEP incidence between those who did and did not receive PEPX (0% vs 0.2%, respectively). Even across all procedures with low post-ERCP risk, there was no significant difference in PEP incidence between those who receive PEPX and those who did not (0% vs 0.2%, respectively). In the high pre-ERCP risk but low post-ERCP risk group, PEP incidence did not differ significantly based on whether PEPX was administered (0% vs 0.8%, respectively). For procedures with both high pre- and post-ERCP PEP risk, patients who received PEPX with PPS alone had a significantly lower PEP incidence (3.2%) than those who received both PPS and RSNAIDs (13.3%; *P* < .001). There was no significant difference in PEP after procedures with low pre-ERCP but high post-ERCP PEP risk between those with no PEPX (5.0%) versus those with any PEPX (0.5%; *P* = .160).

## Discussion

Traditionally, PEP risk assessment has considered operator-, patient-, and procedure-related factors. The results of this study suggest a paradigm shift to a SAFER strategy such that procedure-related factors confer the primary risk, modify patient-related PEP risk, and should drive the decisions regarding PEPX.

An inherently attractive preprocedure strategy is to consider patient-related factors as their risk profile can be conceptualized before performing an ERCP. It is also useful to know that some preprocedure variables are associated with low PEP risk, including chronic obstructive pancreatitis, pancreatic head mass, and prior sphincterotomy. Evaluation of patient-related risk is perhaps most important when deciding if an ERCP should be done and/or by whom, potentially avoiding unnecessary and dangerous diagnostic and empiric procedures.^18^ For patients with traditionally high PEP risk (eg, those with suspected SOD or a history of acute pancreatitis),^11^ careful consideration of procedure necessity, as well as center and operator expertise, is warranted.

Data regarding some potential preprocedure patient-related risk factors, such as young age and female sex, are conflicting. Young females were reported to be high risk for PEP in early studies, when ERCP was more frequently used in patients with suspected SOD.^1^^,^^19^ However, young age and female sex were not risk factors for PEP in the study of a very large national inpatient database from which ERCPs were mostly performed for biliary stones and/or other causes of biliary obstruction and rarely (<10%) for suspected SOD.^9^

Some patient-related anatomical features are only discovered during ERCP. For example, papillary features such as small^20^ or hooded papillae^21^ and are associated with an elevated PEP risk, but cannulation difficulty with varied papillary anatomy is a potential confounding variable. Nearly 50 years ago it was reported that patients with a patent duct of Santorini had less hyperamylasemia and postulated a reduced risk for PEP.^22^ In an RCT of PPS versus sham in the setting of pancreatic SOD, PEP in the sham arm was only observed in patients with a nonpatent Santorini (43%).^23^ In our study, no cases of PEP occurred when the accessory papilla was obviously patent; the incidence of PEP was >6% when it was not patent.

Our data support the concept that PEP risk is primarily linked to procedure-related technical factors specific to the pancreas. PEP pathophysiology is complex, involving chemical and mechanical insults in the setting of impaired papillary drainage, often due to papillary instrumentation or injury from PD contrast injection.^24^^,^^25^ A key pathophysiologic mechanism is procedure-induced papillary edema causing increased PD pressure.^18^ A recent review of PEP prediction models emphasized the important role of intraprocedural factors, such as PD injection, difficult cannulation, precut sphincterotomy, and papillary balloon dilation.^26^

The incidence of PEP in our study for procedures without pancreatography was <1% compared with 4% for those with inadvertent pancreatography. Pancreatography (the number and extent of injections) is widely reported to be a major risk factor for PEP.^19^^,^^27^, ^28^, ^29^, ^30^ One RCT showed that RNSAIDs offered no benefit over placebo for patients without inadvertent pancreatography because of a low overall PEP risk (<5%).^31^ The impact of inadvertent PD manipulation on PEP risk is complex, and depends on the manner and degree of manipulation. For example, in patients with difficult cannulation, early precut sphincterotomy (performed by experienced endoscopists) was associated with lower PEP rates compared with repeated cannulation attempts with the potential for inadvertent PD cannulation, even in patients without PEPX.^32^^,^^33^

Primary wire-guided cannulation, as used in this study, allows for selective deep duct access while reducing papillary trauma and pancreatography risk.^34^ Compared with contrast-assisted cannulation (CAC), guidewire-assisted cannulation (GAC) is known to reduce PEP, especially when managed by experienced endoscopists, and is independent of inadvertent PD cannulation.^35^ Operator-controlled wire-guided cannulation has been available for more than 20 years^36^ and offers advantages by optimizing both the timing and tactile aspects of cannulation.^37^ It is recommended that an experienced operator or assistant control the guidewire, because skillful wire manipulation depends on interpreting tactile feedback to avoid duct injury.^13^

With the increased use of GAC, double-wire cannulation, and transpancreatic precut techniques, concerns about mechanical injury from inadvertent PD guidewire cannulations have been raised.^38^ Studies have reported increased risk of PEP with PD guidewire access during cannulation attempts^39^^,^^40^; however, confounding variables such as concomitant pancreatography, varied GAC-related techniques, and operator expertise were not addressed. An early RCT reported a significantly lower rate of PEP after operator-controlled (2.8%) as compared to assistant-controlled (9.3%) wire-guided cannulation—a finding that was independent of inadvertent pancreatography and PD guidewire cannulation.^41^ Alternatively, PEP may primarily be related to difficult cannulation attempts rather than the PD guidewire as long as guidewire force is minimized to avoid side-branch perforation.^42^ A RCT comparing GAC to CAC reported no cases of PEP among 27 patients after inadvertent PD guidewire cannulation using the GAC technique, whereas the PEP incidence was 19% in those with inadvertent pancreatography using CAC.^43^ Another RCT reported low PEP risk after early precut following 1 or 2 inadvertent PD guidewire cannulations (without contrast) when performed by an expert endoscopist.^44^

In our study, the PEP risk after inadvertent PD guidewire was low, likely due to limited number of guidewire cannulations and/or avoidance of pancreatography. For difficult cannulations deemed unlikely to succeed after a few PD guidewire cannulations, a precut sphincterotomy with PPS was performed. A prospective trial reported low PEP risk using double-wire techniques, even after up to 4 inadvertent PD guidewire cannulations, as long as pancreatography was minimized.^45^ Recent secondary analyses of PEP risk in preprocedure high-risk patients reported that ≥4 PD guidewire cannulations was significantly associated with PEP,^46^ but the beneficial effect of PPS was independent of technical factors.^47^ Potential confounding variables included >100 endoscopists with variable experience, varied cannulation techniques, and >80% of procedures were considered to involve difficult cannulation that more commonly involved trainees. International guidelines recommend PPS for difficult cannulations involving repeated inadvertent PD access, double-wire technique, or precut sphincterotomy.^11^^,^^12^^,^^48^ The American Society for Gastrointestinal Endoscopy PEP guideline also recommends PPS for high-risk scenarios, as long as PD access has been achieved or can be accomplished easily.^13^ Notably, PPS placement failure can occur in nearly 20% of cases,^49^ with PEP risk reaching 30% to 35% following a failed attempt^50^^,^^51^; in such cases, pharmacologic prophylaxis may ameliorate PEP risk.^52^

Impaired PD drainage and increased hydrostatic pressure are likely the key pathophysiologic factors that cause PEP. Early sphincter of Oddi manometry studies demonstrated the role of hydrostatic pressure in PEP. Incidence of PEP was significantly lower when an aspirating catheter was used to decompress the PD.^53^ After biliary manometry only without pancreatic evaluation, PEP did not occur.^54^ Patients with pancreatic SOD were 10 times more likely to develop PEP after manometry testing than those without pancreatic SOD.^55^

Considering the causal PEP factors, most PEP prevention strategies have focused on augmenting PD drainage.^56^ Meta-analyses reported that PPS is effective toward reducing PEP (5%-7%) in high-risk patients.^57^, ^58^, ^59^, ^60^ Compared with RNSAIDs, aggressive hydration, and combinations thereof, PPS was superior in preventing PEP among high-risk patients.^61^ Among both average- and high-risk patients, it was reported that PPS, but not RNSAIDs, significantly reduced moderate to severe PEP.^62^ A recent RCT in high-risk patients reported significantly lower PEP rates and no PEP-related mortality with PPS plus RNSAIDs (11%) compared with RNSAIDs alone (15%).^49^ After transpancreatic precut with PPS in a low-volume center, the incidence of PEP was <4%.^63^ Rescue ERCP with new or exchanged PPS has been shown to effectively treat early PEP, resulting in rapid symptom resolution.^64^, ^65^, ^66^

Our study has several limitations. First, we did not classify papilla morphology in patients with native papillae; certain papilla types are associated with increased cannulation difficulty and higher PEP risk as described. Second, all procedures were performed by a single endoscopist, which may limit the generalizability of our findings. However, data from a single operator allow for consistency related to strategies and techniques. Finally, most PEPX decisions were made at the time of ERCP by the endoscopist, although some were mandated by prospective multicenter trials.

With a SAFER approach, PEP risk may be adjudicated by procedure-related findings and/or events, allowing for individualized PEPX decisions. Other studies have proposed a dynamic risk stratification model that include both patient- and procedure-related factors.^67^^,^^68^ A Japanese study reported that among patient-related risk factors, only native papilla (not young age or female sex) predicted PEP, whereas procedure-related factors (eg, PD guidewire technique, difficult cannulation, PD injection, and absence of PPS) were predictive.^69^ Validated post-ERCP PEP predictive models have shown the best performance in PEP risk assessment.^70^

Our data suggest that PEP risk is primarily influenced by procedural events, and that a SAFER algorithm allows direction of PEPX according to a stratified risk assessment (Fig. 2). High preprocedure risk factors can be downgraded to low risk if pancreatic cannulation and/or pancreatography are avoided. Risk mitigation with PPS should be considered in all cases with increased procedural risk. Scenarios where RNSAIDs are clearly indicated include a history of PEP, inadvertent pancreatography (eg, common channel with filling of PD after bile duct injection), and failed PPS placement. Routine nonselective administration of RSNAIDs in all patients undergoing ERCP has become an axiomatic principle.^71^ Several recent studies have reported acceptably low PEP without RNSAIDs.^63^^,^^72^^,^^73^ Although it has been suggested that both RNSAIDs and PPS should be used in high-risk patients,^49^ our results are consistent with prior data that PPS alone may be sufficient. We found that PEPX can be safely avoided in patients with low post-ERCP risk. This study suggests that universal PEPX is not necessary; a stratified approach that includes procedure-related factors and findings should primarily drive PEPX decisions.

**Figure 2:**
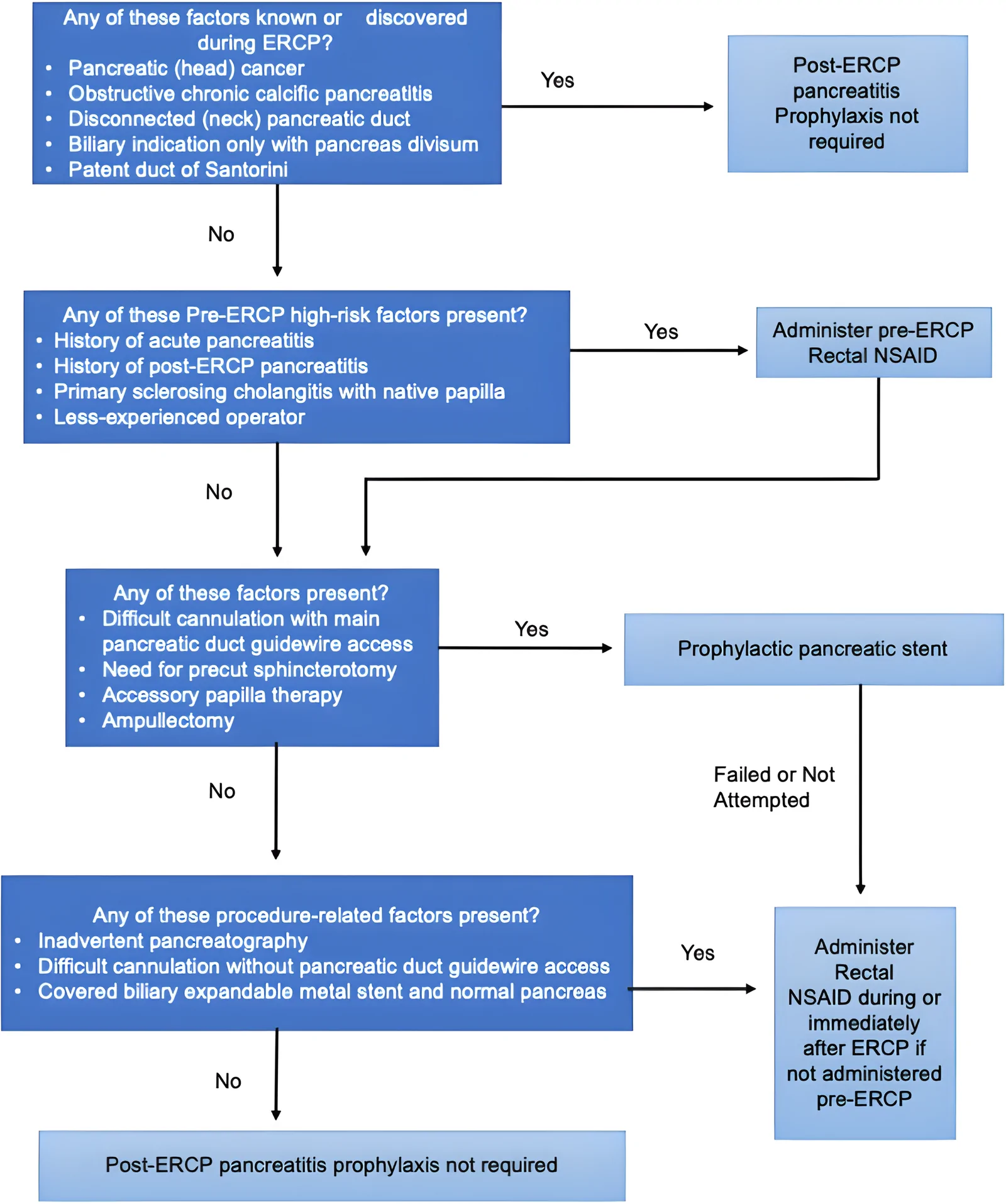
Post-ERCP pancreatitis: stratified assessment for evaluation of risk algorithm. *ERCP*, Endoscopic retrograde cholangiopancreatography; *NSAID*, nonsteroidal anti-inflammatory drug.

## Patient consent

This article does not discuss individual patients; therefore, consent was obtained.

## Disclosure

All authors disclosed no financial relationships.
